# Stable genetic structure and connectivity in pollution-adapted and nearby pollution-sensitive populations of *Fundulus heteroclitus*

**DOI:** 10.1098/rsos.171532

**Published:** 2018-05-09

**Authors:** Joaquin C. B. Nunez, Leann M. Biancani, Patrick A. Flight, Diane E. Nacci, David M. Rand, Douglas L. Crawford, Marjorie F. Oleksiak

**Affiliations:** 1 Rosenstiel School of Marine and Atmospheric Science, University of Miami, 4600 Rickenbacker Causeway, Miami, FL 33149, USA; 2 Department of Ecology and Evolutionary Biology, Brown University, 80 Waterman Street, Box G, Providence, RI 02912, USA; 3 Population Ecology Branch, Atlantic Ecology Division, Office of Research and Development, US Environmental Protection Agency, 27 Tarzwell Drive, Narragansett, RI 02882, USA

**Keywords:** genetic variation, mtDNA, SNP, microsatellites, population genetics, pollution cline

## Abstract

Populations of the non-migratory estuarine fish *Fundulus heteroclitus* inhabiting the heavily polluted New Bedford Harbour (NBH) estuary have shown inherited tolerance to local pollutants introduced to their habitats in the past 100 years. Here we examine two questions: (i) Is there pollution-driven selection on the mitochondrial genome across a fine geographical scale? and (ii) What is the pattern of migration among sites spanning a strong pollution gradient? Whole mitochondrial genomes were analysed for 133 *F. heteroclitus* from seven nearby collection sites: four sites along the NBH pollution cline (approx. 5 km distance), which had pollution-adapted fish, as well as one site adjacent to the pollution cline and two relatively unpolluted sites about 30 km away, which had pollution-sensitive fish. Additionally, we used microsatellite analyses to quantify genetic variation over three *F. heteroclitus* generations in both pollution-adapted and sensitive individuals collected from two sites at two different time points (1999/2000 and 2007/2008). Our results show no evidence for a selective sweep of mtDNA in the polluted sites. Moreover, mtDNA analyses revealed that both pollution-adapted and sensitive populations harbour similar levels of genetic diversity. We observed a high level of non-synonymous mutations in the most polluted site. This is probably associated with a reduction in *N*_e_ and concomitant weakening of purifying selection, a demographic expansion following a pollution-related bottleneck or increased mutation rates. Our demographic analyses suggest that isolation by distance influences the distribution of mtDNA genetic variation between the pollution cline and the clean populations at broad spatial scales. At finer scales, population structure is patchy, and neither spatial distance, pollution concentration or pollution tolerance is a good predictor of mtDNA variation. Lastly, microsatellite analyses revealed stable population structure over the last decade.

## 1. Introduction

Worldwide, anthropogenic activities have resulted in drastic ecosystem changes [1] and introduced novel stressors to natural populations. Populations of the marine teleost *Fundulus heteroclitus* have adapted to anthropogenic contaminants, and embryos from populations inhabiting heavily polluted estuaries show heritable resistance to persistent organic pollutants [2]. This resistance is not found in embryos from populations inhabiting relatively uncontaminated areas [2–6]. To investigate signatures of population structure and natural selection in pollution-adapted *F. heteroclitus* populations, we used mitochondrial sequences (mtDNA) and microsatellites to compare pollution-adapted *F. heteroclitus* inhabiting the highly polluted New Bedford Harbour (NBH) estuary to neighbouring pollution-sensitive populations in southern Massachusetts. NBH has been contaminated since 1940 [7] and was designated an Environmental Protection Agency (EPA) Superfund site in 1982. This site contains high levels of persistent, bioaccumulative contaminants including polychlorinated dibenzodioxins (PCDDs), polychlorinated biphenyls (PCBs) and polycyclic aromatic hydrocarbons (PAHs) [4]. Adaptation to anthropogenic pollutants is often associated with the survival of individuals with high tolerance phenotypes [8], and in the case of *Fundulus* these adaptive responses to contaminants occurred rapidly [2,6,9–11]. Moreover, Reid *et al*. [12] showed that pollution-adapted populations living in the most polluted area of the NBH estuary were independently derived from the local gene pool of non-adapted populations (i.e. adapted from standing genetic variation). This process reduced the polluted population's effective population size (*N*_e_) relative to neighbouring unpolluted areas. The fish evaluated by Reid *et al*. [12] were collected from the uppermost, most polluted, area in the New Bedford estuary (sediment pollution 22 000 ng g^−1^ dry weight (dw) of PCB_126_). However, the polluted habitat in NBH extends at least 5 km south of this collection site, with sediment pollution levels decreasing outwards towards Buzzards Bay, where pollution remains around 541 ng g^−1^ dry weight of PCB_126_, and *Fundulus* populations inhabit the entire stretch of polluted estuary (hereby referred to as pollution cline). Work by Nacci *et al.* [3] has shown that, fish inhabiting different habitats along the pollution cline are exposed to different PCB_126_ levels depending on their local habitat, and show differential survival (LC50; table 1). Additionally, recent work by Du [13] has shown that, for populations across NBH's pollution cline, allele frequencies of nuclear loci covary significantly in response to the concentration of sediment pollution. These studies illustrate that genetic responses to pollution in NBH are proportional to polluting agent concentration.

**Table 1.** Location, PCB_126_ concentration, and LC50 of each collection site used for the mtDNA analysis of the pollution cline.

location	population group	acronym	latitude	longitude	PCB in sediment (ng g^−1^ dw)	LC50 ng PCB 126 l^−1^	coastal distance to NBH (km)
New Bedford Harbour, MA	pollution-adapted	NBH	N 41°40′ 19.2″	W 70°54′ 50.4″	22 667	29 174 270	0
Sycamore Street, New Bedford, MA	pollution-adapted	SYC	N 41°39′ 14.4″	W 70°54′ 54.0″	3760	96 383	2.4
Pilgrim Avenue, New Bedford, MA	pollution-adapted	PA	N 41°38′ 45.6″	W 70°54′ 35.9″	874	250 035	3.3
Fairhaven Launch, Fairhaven, MA	pollution-adapted	FL	N 41°37′ 55.2″	W 70°54′ 14.4″	541	488 652	4.27
Hacker Street, Fairhaven, MA	pollution-sensitive	HS	N 41°37′ 51.6″	W 70°52′ 55.2″	13	1127	5.1
Slocums River, Westport, MA	pollution-sensitive	SR	N 41°32′ 9.60″	W 70°58′ 11.9″	7	281	31.2
Mattapoisett, MA	pollution-sensitive	MA	N 41°38′ 9.60″	W 70°51′ 57.6″	27	205	29.0

As a species, *F. heteroclitus* exhibits remarkably limited home ranges and dispersal estimates of less than 1 km [14,15]. This life-history trait may result in fine-scale population structure at the microhabitat level (e.g. [16]). Pollution in NBH continues to act as a strong environmental stressor, and studies have shown that individuals from pollution-adapted populations display altered metabolic processes relative to pollution-sensitive fish [17,18]. Since mtDNA encodes 13 proteins involved in the oxidative phosphorylation pathway (OXPHOS), mtDNA mutations conferring differential fitness in polluted environments may become targets of natural selection. This effect of selection could be indirect, related to energetic costs of metabolizing the pollutants, or from the pollutants directly affecting OXPHOS function. We test the general hypothesis that selection has increased the frequency of specific mtDNA haplotypes in pollution-adapted versus pollution-sensitive populations. Our null hypothesis is no significant reduction of variation across sites. Moreover, we characterized putatively functional amino acid changes in the 13 mtDNA-encoded OXPHOS proteins between pollution-adapted and pollution-sensitive fish.

**Figure 1.**
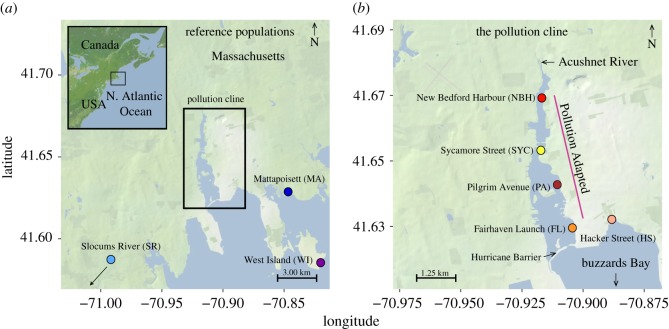
Collection sites. (*a*) Reference populations flanking the pollution cline. Samples from MA and SR were collected in 2014 and were used as reference for the mtDNA experiments. The population from WI was used as reference for the microsatellite study and was sampled in 1999 and 2007. (*b*) Sites within the pollution cline. The population in NBH was sampled three times: 2014, 2008 and 2000. Samples from 2008 and 2000 were used in the microsatellite analysis; samples from 2014 were used in the mtDNA analysis.

In addition, we used mtDNA sequences to characterize population structure and connectivity between and among pollution-adapted and pollution-sensitive populations. We collected fish from five sites distributed along the NBH pollution cline (table 1 and figure 1). Four of these sites are inhabited by pollution-adapted individuals. The fifth site, lying outside the hurricane barrier at the mouth of NBH (figure 1*b*), is inhabited by pollution-sensitive individuals. Two additional pollution-sensitive populations were collected approximately 30 km from NBH. Finally, we used microsatellite markers to test the genetic signal's temporal stability over the last decade (2000–2008). Microsatellite analyses were conducted on two populations, one within and one outside NBH, at two time points, 1999/2000 and 2007/2008. Both these markers, mtDNA and microsatellites, are highly sensitive to recent demographic events [19].

## 2. Methods

Complete mtDNAs of *F. heteroclitus* were reconstructed from raw reads from the National Centre for Biotechnology Information (NCBI) Short Read Archive (SRA) BioProject ID: PRJNA291654 published in [20]. These samples correspond to fish collected from seven locations in Massachusetts (table 1) in 2014. Four of these sites (NBH, SYC, PA, FL; figure 1*b*) lie on a steep pollution cline between 1 and 3 km of each other (by coastal distance); the remaining three (HS, SR, MA; figure 1*a*) lie in adjacent, relatively unpolluted habitats [3]. Fish inhabiting polluted sites (NBH, SYC, PA, FL) have been shown to be well adapted to pollution based on laboratory experiments [3]. The same experiments also showed that fish from HS, SR and MA display reduced pollution resistance. Thus, in this analysis, we refer to individuals from NBH, SYC, PA and FL as ‘pollution-adapted’ fish. Conversely, fish from the remaining three sites are referred to as ‘pollution-sensitive’. Samples for the microsatellite analysis were collected from West Island, Massachusetts (WI; a clean site; figure 1*a*) and NBH sites in 2007 and 2008, respectively, using minnow traps and seine nets. Tissue samples from historical populations (WI-1999 and NBH-2000) were provided by the US EPA Atlantic Ecology Division. DNA for microsatellite analyses was extracted from 30 fish each from the NBH and WI populations using the animal tissue spin-column protocol of a Qiagen Dneasy® Tissue Kit. Eight oligonucleotide primer pairs designed for the amplification of trinucleotide ATG microsatellite repeats were chosen from Adams *et al.* [21]. Forward primers were labelled with one of three fluorescent dyes (HEX, 6-FAM and NED). Each primer pair was used to amplify each DNA sample in a separate 25 µl polymerase chain reaction (PCR) consisting of 1 µl DNA, 2.5 µl 10× buffer, 1 µl forward primer, 1 µl reverse primer, 1 µl of 0.2 µM dNTPs, 0.1 µl Taq DNA polymerase and 18.4 µl Milli-Q water. Thermal cycling conditions consisted of 94°C for 2 min followed by 35 cycles of 94°C for 30 s, 54°C for 30 s, 72°C for 75 s and 72°C for 10 min. PCR products were electrophoresed on 1% agarose gels to confirm amplification of DNA samples and blank negative controls.

Genotyping at eight microsatellite loci was conducted at the Centre for Applied Genomics at the Hospital for Sick Children in Toronto, Canada. A mutation step consisting of one nucleotide base pair was discovered post-genotyping at the FhATG-17 locus. This was assumed to be an error because the most common allele at FhATG-17 consists of a 135 bp fragment and the incompatible mutation step (which yielded a 136 bp fragment) was only present in three individuals, one of which was a homozygote. It is very unlikely that such an extremely rare allele would exist as a homozygote and, therefore, the four 136 alleles were changed to 135. Fragment lengths flanking the trinucleotide repeats were determined for each locus by aligning primer sequences with sequence data made available by Adams *et al.* [21] through GenBank.

Genome estimates of nucleotide diversity (*π* = *K*/*L*, where *L* is the sequence length and *K* is the estimated average number of nucleotide differences [22]), segregating sites (*S*), haplotype number (*h*) and haplotype diversity (*h*_d_) were conducted using DNAsp v. 5.10.1 [23]. Population differentiation estimates (*F*_ST_) were conducted in Arlequin v. 3 [24] with 1000 permutations, 100 000 steps in the Markov chain and 10 000 de-memorization steps. Migration estimates were obtained using a coalescent framework implemented in MIGRATE v. 3.6.11 [25] using a Bayesian inference strategy. Similar to the approach taken by Duvernell *et al*. [15], all MIGRATE analyses were conducted using a series of pairwise analysis assuming a stepping-stone model [26] (electronic supplementary material, figure S1). This model was chosen acknowledging the life history of *F. heteroclitus.* For all pairs of populations compared, we asked whether a panmixis model (i.e. the two collection sites, *i* and *k*, separated by distance *d_ki_* came from the same population), or a stepping-stone model (i.e. two collection sites are discrete demes with asymmetric migration), would better fit the data. All analyses were conducted using uniform priors for both theta (*Θ*) and *M_k _*_→ _*_i_* (where *M_k _*_→ _*_i _*= *m/μ*, migration from population *k* to population *i* is given in terms of *m*, the migration rate, and *μ* is the mutation rate). We used complete mtDNA sequences as input using the ‘sequence data’ option. This parameter ensures that the input is read as haplotypes (i.e. SNPs are not considered independent). Runs were performed using four heated chains (with temperatures as suggested by the MIGRATE manual; 1.00, 1.50, 3.00, 1 × 10^6^; swapping interval = 1). Runs were allowed a burn-in of 1 × 10^5^ steps, and the long chains ran with 5 × 10^5^ steps. Run convergence was assessed using effective sample size metrics (ESS). Model evidence was compared based on marginal likelihoods (mL) using a Bezier curve approximation (see the MIGRATE manual for details on the method). Statistical analyses were done in R v. 3.3.2 [27] using tools from the tidyverse suite v. 1.2.1 [28,29]. Statistical parsimony networks were reconstructed using complete mtDNA haplotype sequences using PEGAS v. 0.10 [30]. AMOVA analyses were calculated using the package ade4 in R [31]. Isolation by distance (IBD) analyses were done in adegenet (v. 2.1.1) [32]. Principal component analyses (PCA) were done in FactoMineR v. 1.39 [33] and visualized with factoextra v. 1.0.5. Variance partitioning analyses (VPA) were done using Vegan v. 2.4-5 [34], considering three predictor variables: geographical distance, sediment pollution (ng g^−1^ dry weight of PCB_126_) and pollution tolerance (LC50 to PCB_126_); these data were obtained from Nacci *et al.* [3]. The *F. heteroclitus* mitochondrial genome is fully mapped in the NCBI database; thus, SNPs were mapped to genes by direct association to the database. Protein topology analyses were done using TOPCONS [35]; tRNA secondary structure analyses were predicted using tRNA scan-SE [36], ARWEN [37], the rnafold function in Matlab® and visualized in VARNA v. 1.0.1 [38]. Microsatellite analyses were conducted on the Microsatellite Analyser (MSA) v. 4.05 software [39]. Pairwise and global *F*_ST_ values were calculated in MSA with 100 000 data permutations. MSA was also used to estimate measures of genetic variation including theta (*θ*) and expected heterozygosity (*H*_E_).

## 3. Results

### 3.1. Mitochondrial genome mapping

Mitochondrial genomes from 192 *Fundulus heteroclitus* samples [20] were mapped to a reference genome using Bowtie2 [40]. Any sequence showing more than 1% missing data (i.e. proportion of ambiguous bases labelled as ‘N’) was removed from the analysis. Thus, we analysed 133 sequences across all populations (NBH: 22, SYC: 17, PA: 24, FL: 12, HS: 18, MA: 18, SR: 22). The average coverage of the consensus alignments was 75-fold and the mean mapping quality was 36.9. The average GC content of the genome was 41.98%, and the total genome size was 16 527 bp. However, 2625 sites spaced throughout all 133 samples contained gaps or missing data (N) in at least one individual and were not used for analysis by DNAsp. On average, 14 sites per individual were called as heteroplasmic (A/T, C/G, A/C, G/T, A/G or C/T). The range of heteroplasmic sites varied from 54 to 0 sites per sequence with A/G and C/T as the most abundant calls. It is difficult to accurately differentiate truly heteroplasmic loci from sequencing errors, or from mixed populations of cells homoplasmic for each variant; thus, all loci with more than two variants within an individual were relabelled as ‘N’. Among all used sites in all populations, 13 424 were monomorphic and 478 sites in the alignment were polymorphic; 327 of the polymorphic sites were singletons across the entire dataset and 151 were parsimony informative variable sites. None of the variable sites were fixed in any sub-group or population. Singletons in the dataset were excluded from all analyses. These sites are problematic due to the difficulty in discerning ‘real’ singletons from PCR or sequencing errors and conserving these sites may introduce strong biases to the dataset [41]. It is important to note that we only eliminated singleton SNPs observed only once throughout the entire dataset. SNPs that appear as singletons in subdivisions of the dataset, such as individual populations or population groups (e.g. pollution-adapted or pollution-sensitive sites), were kept. Removal of singletons weakens the power of neutrality statistics that depend on the site frequency spectrum (e.g. Tajima's *D*) [41]; as such, we refrained from using these kinds of statistics on our data.

### 3.2. Mitochondrial estimates of genetic diversity and differentiation

Estimates of mtDNA genetic diversity are shown in table 2. Since 2952 sites were excluded in DNAsp due to alignments gaps or missing data, our estimates may slightly underrepresent the genetic variation in the populations. However, this conservative approach ensures that the variation estimates are calculated using markers consistently represented in all samples. The average number of observed SNP differences (*K*) is similar in both groups (*K*_adapted _= 8.644, *K*_sensitive _= 7.623). Individual estimates of *K* for each population were close to the group's average with NBH (the most polluted population) showing the highest *K* (9.069), and HS the lowest (*K* = 6.052). Haplotype diversity (*h*_d_) [22] is high among all samples (table 2) and we observed 77 haplotypes across all sites (*h*_d general_ = 0.985 ± 0.004). MtDNA nucleotide diversity (*π*) estimates for adapted and sensitive populations were within the standard error (s.e.) of each other (*π*_adapted _= 0.064 ± 0.004%, *π*_sensitive _= 0.056 ± 0.005%). Site-specific *π* values showed similar diversity levels relative to the mean group *π* and to each other. We also report *π* for mitochondrial functional genes: subunits 1, 2, 3, 4 and 4 L of the *NADH dehydrogenase* gene (*ND*), subunits I, II and III of the *Cytochrome oxidase* gene (*COX*), the *Cytochrome B* gene (*CytB*), subunits 6 and 8 of the *ATP-synthase* gene (*ATP*) and non-protein-coding genes rRNA-12S and rRNA-16S. The D-loop is also shown (electronic supplementary material, table S1). The D-loop is a highly variable region, and some of the variant sites were excluded from the alignment due to a high level of singletons and missing nucleotides (N) not seen in other mtDNA regions. As a result, our *π* estimates for the D-loop probably underestimate the real nucleotide diversity of the populations. Overall, 91 SNPs are shared between both population groups; 36 SNPs are polymorphic in the pollution-adapted populations and monomorphic in the pollution-sensitive populations, and 24 SNPs are polymorphic in pollution-sensitive populations and monomorphic in pollution-adapted ones. Pairwise comparisons between adapted and sensitive populations resulted in an *F*_ST_ value for mtDNA of 0.0299 (*p* < 0.001). Pairwise *F*_ST_ analyses of inter-population (between collection sites) differentiation are shown in table 3. With the exception of comparisons with SR, most *F*_ST_ values were low (mean *F*_ST_ = 0.019, s.d. = 0.015) and statistically non-significant. Additionally, comparison of HS versus NBH, and HS versus MA also showed statistical significance (*p* < 0.05). Analyses of molecular variance (AMOVA) between the pollution-adapted and pollution-sensitive populations revealed that most of the genetic variation was found within the individual sites (97%; Monte Carlo test *p* < 0.05; 50 000 replications). Other sources of genetic variance, variation among grouped sites (1%) and between the groups (2%), contributed marginally to the global variance.

**Table 2.** Various genetic diversity measures: *n*_ind_, sample size; S, segregating sites; *h*, number of unique haplotypes; *h*_d_, haplotype diversity; *K*, average number of differences; *π*, nucleotide diversity. Only unique SNPs were considered when reporting diversity estimates in population groups and the standard error (s.e.). These measures were calculated using an alignment of genomes collected from all seven sites. In this alignment, nucleotide sites with mismatches or gaps were excluded.

population	*n*_ind_	*S*	*h*	*h*_d_	*K*	*π* (±s.e.)
NBH	22	58	15	0.9614	9.07	0.067% (0.008%)
SYC	17	59	15	0.9853	9.00	0.066% (0.009%)
PA	24	64	17	0.9377	7.67	0.057% (0.008%)
FL	12	41	11	0.9848	8.61	0.063% (0.009%)
HS	18	44	14	0.9542	6.05	0.045% (0.006%)
SR	22	45	17	0.9653	7.85	0.058% (0.007%)
MA	18	55	16	0.9869	8.11	0.060% (0.007%)
adapted group	75	127	43	0.9770	8.255	0.056% (0.004%)
sensitive group	58	15	43	0.9846	7.623	0.064% (0.004%)

**Table 3.** Below the diagonal: within population pairwise *F*_ST_ values calculated from mtDNA data. Above the diagonal: corresponding *F*_ST_ *p-*values. Negative *F*_ST_ values are reported as ∼0.00.

		*p*-value						
comparison		NBH	SYC	PA	FL	HS	SR	MA
*F*_ST_	NBH	*	0.21	0.09	0.10	< 0.001	< 0.001	0.28
	SYC	0.01	*	0.17	0.14	0.31	< 0.001	0.88
	PA	0.02	0.01	*	0.93	0.11	< 0.001	0.16
	FL	0.03	0.02	∼0.00	*	0.19	< 0.001	0.19
	HS	0.06	∼0.00	0.02	0.01	*	< 0.001	0.01
	SR	0.07	0.04	0.06	0.05	0.06	*	0.01
	MA	0.01	∼0.00	0.01	0.02	0.03	0.04	*

### 3.3. Mitochondrial haplotype analyses

**Figure 2.**
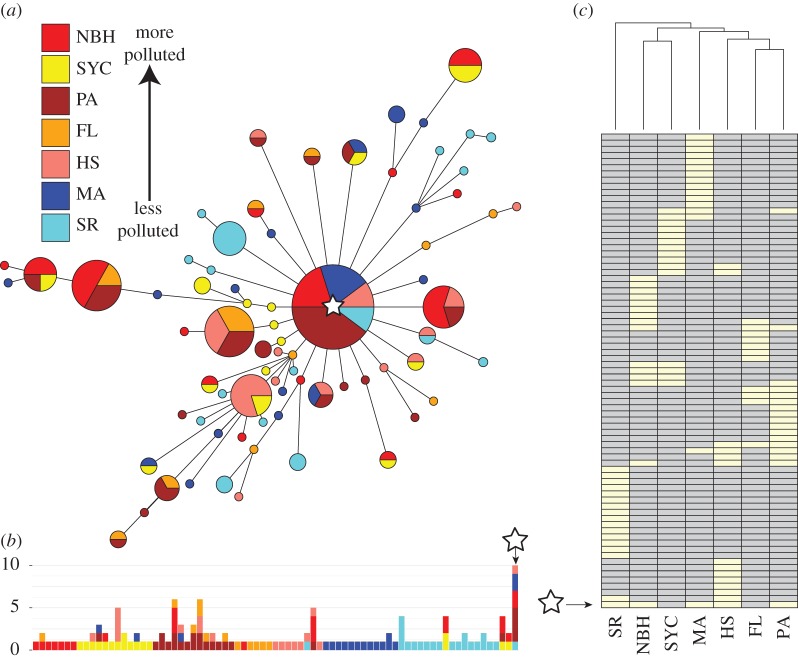
(*a*) Maximum-parsimony haplotype network considering all haplotypes in the sample. (*b*) Distribution of haplotypes among all 133 samples. The abscissa represents each of the 77 haplotypes in the sample. The stacked bars represent the number of individuals showing that particular haplotype colour-coded by population. (*c*) Shared haplotype matrix. Each row in the matrix represents each of the 77 haplotypes. Light/dark boxes represent the presence/absence of that haplotype in a particular population. In all cases, the star indicates the most common haplotype.

**Figure 3.**
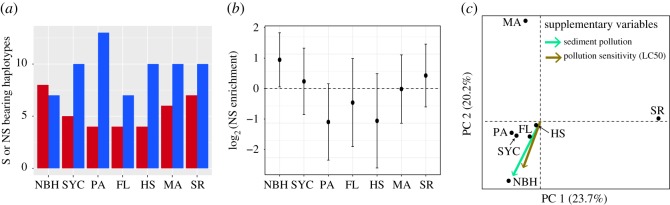
(*a*) Frequency of haplotypes bearing at least one non-synonymous SNP (NS; red bars), and haplotypes with only synonymous SNPs (S; blue bars), across all populations. (*b*) Relative enrichment (odds ratio) of NS relative to S across populations. (*c*) Principal component analysis in mtDNA haplotype frequencies. Sediment pollution (PCB_126_ sediment concentration; green arrow) and pollution sensitivity (LC50 to PCB_126_; brown arrow) were added into the PCA as supplementary variables.

A haplotype network constructed using the infinite sites model reveals the presence of a major haplotype at high frequency across all sites except FL and SYC (figure 2*a,b*). All other haplotypes radiate in a star-like fashion out from the major haplotype. This is consistent with the known historical phylogeography of *Fundulus* [15]; 75% (58) of all haplotypes are unique to each population, 15% (12) are shared between two populations and 8% (6) are shared among three populations. Clustering analysis based on shared haplotypes showed that SR is the most dissimilar of all populations (figure 2*c*). The most polluted habitats, NBH and SYC, share the most haplotypes. In addition, NBH showed an excess of haplotypes containing at least one non-synonymous SNP (figure 3*a*). When considering the total number of segregating SNPs, NBH harbours a higher proportion of non-synonymous mutations segregating across its private haplotypes (figure 3*b*). PCA on haplotype frequencies among all sites is shown in figure 3*c*. Jointly, the first two principal axes explained 44% of the variance. The first principal component isolates the SR population from all other populations. The second principal component primarily isolates the MA population from the inhabitants of the pollution cline. Amounts of sediment pollution (PCB_126_), and LC50 were added to the PCA as supplementary variables**.** We also conducted a second PCA with the cline populations (electronic supplementary material, figure S2). PC 1 of this analysis (32% variation explained) strongly segregated the most polluted populations (i.e. NBH and SYC) from the rest of the cline. Factor loadings for PC 1 showed that three haplotypes had strong positive correlations with populations in the most polluted sites (*r* > 0.98, *p* < 0.003). Sixteen additional haplotypes showed positive correlations between 0.56 and 0.70. The supplementary variables, sediment PCB_126_ and LC50, showed correlations of 0.73 and 0.63, respectively.

### 3.4. Gene flow and spatial analyses (broad- and fine-scale patterns)

**Figure 4.**
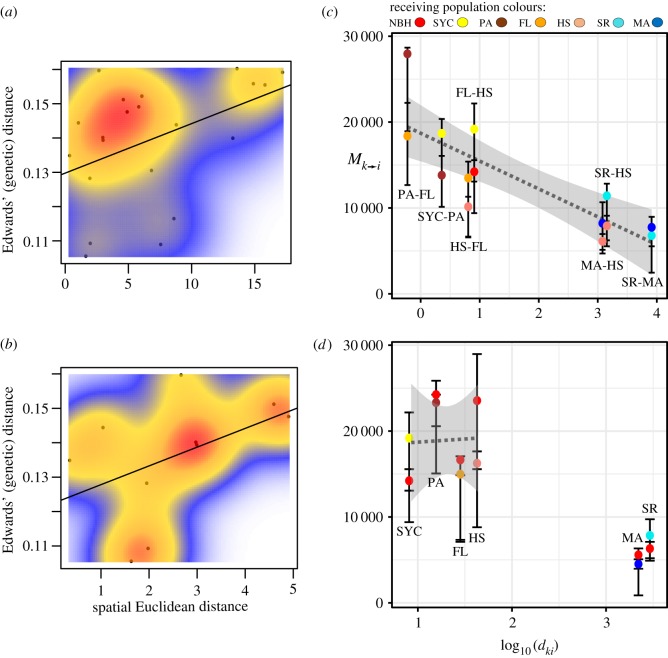
Spatial patterns and migration rates. (*a*) IBD analysis considering all seven populations (i.e. broad scale). (*b*) IBD analysis considering only the populations within the cline (i.e. fine scale). (*c*) Migration rates along the pollution cline and neighbouring sites. The figure shows median mutation-scaled migration rates *M_k_*_→_*_i_* (as well as the 25th and 75th IQRs of the posterior distribution) relative to log_10_ transformed coastal distances between sites (*d_ki_*). The colours of each data point correspond to the receiving population (*i*). Migration routes are labelled throughout the graph. Linear model and 95% confidence intervals are shown. (*d*) Migration rates estimated in pairwise comparisons of NBH relative to all other populations. Linear model and 95% confidence intervals are shown only for estimates within the pollution cline.

We characterized correlations between genetic distance (estimated from whole mtDNA haplotypes) and predictor variables at two spatial scales: broad scale (all populations) and fine scale (the pollution cline only). At the broad scale, a Mantel test showed a marginally significant non-random association between genetic and spatial distance (Monte Carlo test *p* < 0.06; 50 000 replications). This signature of IBD reveals the existence of two main patches in the data: the pollution cline and MA, and SR (figure 4*a*). If MA is removed from the analysis (see Discussion), the statistical association of IBD strengthens (Monte Carlo test *p* < 0.015; 50 000 replications). Variation partition analysis (VPA) allows estimating the proportion of mtDNA genetic variation explained by a single predictor variable controlling for the other two predictors. At broad spatial scales, geographical distance explained 14.74% of the variation, followed by sediment pollution concentration of PCB_126_ (1.51%) and lastly by LC50 (less than 1%). The remaining variance was residual. These results suggest that IBD drives a portion of mtDNA variation at the broad scale. At the fine scale (cline alone), Mantel tests for IBD were not significant (Monte Carlo test *p* > 0.1; 50 000 replications). The analysis also revealed that the spatial structure of cline populations is patchy (figure 4*b*), and none of the three predictor variables significantly explained mtDNA variation.

We estimated migration rates assuming a stepping-stone model (electronic supplementary material, figure S1). For each pair of populations, we tested whether panmixis or a model with asymmetric migration between two discrete populations would better fit the data. We also estimated migration between NBH and all other populations. For all comparisons, the two-population model outperformed the panmixis model (electronic supplementary material, table S2). Migration rates (*M_k _*_→ _*_i_*) for population pairs across the cline and neighbouring sites ranged from 4500 m/µ to 30 000 m/µ. The analyses showed that migration is high among proximal populations; however, it decreases substantially with respect to distal populations (SR and MA) (*d_ki_*, figure 4*c*; *R*^2^ = 0.6065, *F*-statistic: 24.12, *p* < 0.01). Migration rate estimates for all populations relative to NBH echo this pattern (Figure 4*d*; for NBH versus cline populations (SYC, PA, FL, HS), correlation of migration rates and distance is not significant).

### 3.5. Characterization of non-synonymous mtDNA SNPs

Across all the dataset, 16 (17%) segregating SNPs caused non-synonymous substitutions in mtDNA protein-coding genes (electronic supplementary material, table S3). These SNPs, however, were not consistent with the pattern of environmental pollution. Thus, there is no evidence to suggest that pollution has acted as a selective agent on the mtDNA. We observe non-synonymous substitutions occurring in genes: *ND5*, *ND6*, *ND4*, *ND3*, *ND2*, *ATP6* and *COI* (electronic supplementary material, table S3). The substitution in *COI*, however, is presumed to retain the identity of the initiator codon. Montooth *et al.* [42] identified a change in the initiation codon of *COI* and *ND1* in *Drosophila*. We observed an initiation codon other than AU*R* (*R* = A or G) in the *F. heteroclitus COI* gene (ATC). This initiation codon has been observed in *Mus* [43]; however, it had not been observed in *Fundulus*. All amino acid changing SNPs were called with a sequencing depth of at least ten reads per site [44]. These amino acid changing substitutions did not occur as isolated haplotypes; out of the 77 observed haplotypes, 16 contained either a single or a combination of non-synonymous SNPs. Consistent with other studies [45], predictions of protein topology showed that most of the substitutions affected transmembrane amino acids (electronic supplementary material, figure S3A).

Segregating polymorphisms were also seen in non-protein-coding regions of the mtDNA (electronic supplementary material, table S3B). Nine segregating, non-singleton SNPs were seen in transfer RNAs and mapped to their respective structures. One SNP in tRNA-Ala mapped to the anticodon loop region (electronic supplementary material, figure S3B). Substitutions occurring in rRNA regions showed the highest percentage of individuals with segregating sites in SR (31.8%) followed by FL (33.3%), PA (25.0%), SYC (17.6%), HS (16.6%), MA (11.1%) and NBH (9.1%). Substitutions affecting only tRNAs showed the highest percentage of segregating sites in individuals in PA (33%) followed by FL (33%), HS (27%), NBH (22%), SYC (17.6%), MA (11.1%) and SR (4.5%).

### 3.6. Microsatellite analyses of genetic differentiation

Consistent with our mtDNA analysis, all microsatellite loci of WI-1999, WI-2007, NBH-2000 and NBH-2008 showed very low genetic differentiation among populations, which was not significant (*F*_ST_ = 0.005, *p* > 0.05). Overall, the four samples (two sites at two time points) are not highly differentiated. The global *F*_ST_ value for one locus, FhATG-20 is significant and shows higher differentiation (*F*_ST_ =  0.030, *p* < 0.05). Overall, pairwise *F*_ST_ values between different sites during the same time period (1999/2000 and 2007/2008 populations) are small and statistically non-significant (table 4) thus echoing the general trends from the mtDNA markers. The pairwise *F*_ST_ value between WI-1999 and NBH-2008, however, suggests a difference in the genetic structure for these two populations (*F*_ST_ = 0.024, *p* < 0.01). Pairwise *F*_ST_ value estimates between samples from the same location during different years are low and non-significant suggesting stable genetic structures at either site.

**Table 4.** Below the diagonal: population pairwise *F*_ST_ values calculated from microsatellite data. Above the diagonal: corresponding *F*_ST_ *p*-values. Negative *F*_ST_ values are reported as ∼0.00.

		*p-*value			
comparison		WI-1999	WI-2007	NBH-2000	NBH-2008
*F*_ST_	WI-1999	*	0.35	0.12	<0.001
	WI-2007	0.00	*	0.49	0.47
	NBH-2000	0.01	∼0.00	*	0.77
	NBH-2008	0.024	∼0.00	∼0.00	*

**Figure 5.**
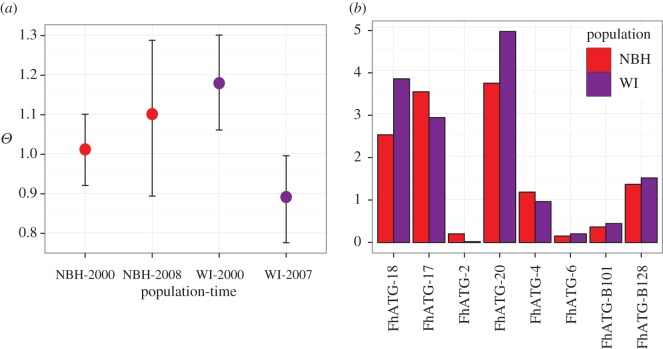
Microsatellite loci analyses. (*a*) Weighted average theta values overall loci from Microsatellite Analyser (MSA, based on gene diversity and stepwise mutation model). Confidence intervals estimated by standard error of weighted theta values across loci. (*b*) Theta estimates for each microsatellite locus with data pooled across years. Theta values are based on gene diversity and stepwise mutation model.

It is unclear whether the differentiation between WI-1999 and NBH-2008 is driven by location or by time (see Discussion). To further explore this problem, independent analyses were carried out pooling the data across sites and across years. When sites are pooled, the global *F*_ST_ value for all loci shows very low differentiation between the archived and recent samples and the *F*_ST_ value is not significant (*F*_ST_ = 0.004, *p* > 0.1). Furthermore, the global *F*_ST_ value at FhATG-20 (the one locus with a significant *F*_ST_ value without pooling) is not significant for pooled sites (*F*_ST_ = 0.006, *p* > 0.1). On the other hand, when time periods are pooled, the global *F*_ST_ value is slightly higher and is significant (*F*_ST_ = 0.009, *p* < 0.05). There is no sign of change in the levels of genetic diversity in the NBH (2000 and 2008) population between the time points surveyed (figure 5*a*). When time periods are pooled, there are a few loci that show reduced diversity in NBH (figure 5*b*). However, with this number of loci it is impossible to assess the significance of these outlying loci. It should be noted that the locus showing the largest diversity reduction in NBH is FhATG-20, the same locus exhibiting a significant *F*_ST_ value.

## 4. Discussion

We sequenced 133 complete mtDNAs from populations along a pollution cline as well as neighbouring unpolluted areas. We examined two questions: (i) Is there pollution-driven selection on the mitochondrial genome across a fine geographical scale? and (ii) What is the pattern of migration among sites spanning a strong pollution gradient? Our results showed no evidence for a selective sweep of mtDNA in the polluted sites. All sites showed similar levels of differentiation at the level of genetic variation between population groups (*π*_adapted _= 0.064 ± 0.004%, *π*_sensitive _= 0.056 ± 0.005%; table 1). Our mtDNA analysis indicated the presence of unique haplotypes significantly correlated with polluted or clean sites. Moreover, the most polluted habitat, NBH, showed an enrichment of non-synonymous SNPs (figure 3*b*). We also characterized population structure and connectivity among populations. This analysis revealed two different population structure dynamics depending on spatial scale. At broad spatial scales, we observed that IBD explained a portion of the population structure. However, at fine spatial scales, distance does not appear to play a significant role. Lastly, based on our analyses of eight microsatellite loci, population structure appears stable over the last decade (figure 5).

### 4.1. Pollution does not cause directional selection on mtDNA

Our analyses did not detect any mtDNA haplotype sweeping through pollution-adapted populations, thus rejecting our hypothesis for directional selection on mtDNA. A possible corollary of this hypothesis is that selection is driving multiple mtDNA haplotypes to fixation, and selective interference of these haploid, non-recombining, mtDNAs is preventing the fixation of any one haplotype. While possible, this seems unlikely because it requires that all competing mtDNA haplotypes show nearly identical fitnesses to be maintained under haploid selection [46]. However, the excess of non-synonymous mutations in the most polluted site (NBH) may be suggestive of weakened purifying selection. Natural selection's ability to fix or purge variation is directly proportional to *N*_e_. Thus, when *N*_e_ is reduced (e.g. after a pollution-related bottleneck), the rate at which deleterious or mildly deleterious mutations are purged is reduced [47]. A second hypothesis is that enrichment for non-synonymous SNPs resulted from the opposite process. That is, demographic expansion following a bottleneck leading to increases in the relative abundance of rare and deleterious variants [48]. Both these hypotheses are compatible with the case of rapid adaptation in NBH described by Reid *et al*. [12]. They differ, however, in the assumptions related to the timing, number and recurrence of bottlenecks in these populations since pollution was first introduced. Interestingly, while SYC, PA and FL also inhabit polluted habitats (figure 1) and show pollution-resistance phenotypes (table 1; [2]), they do not show any signs of population reduction, either at the level of nucleotide diversity, haplotype diversity (table 2), or in their distributions of synonymous versus non-synonymous mutations (figure 3*a,b*). An additional hypothesis can be considered: the high proportion of non-synonymous SNPs in NBH could be explained by increased mutation rates resulting from pollution exposure. This is a possible alternative as recent studies have shown that PCB exposure can induce mutations as well as chromosome breaks [49]. Testing hypothesis related to increased mutation rates is beyond the scope of our current dataset. However, deep sequencing of mtDNAs on fish embryos exposed to varying concentrations of the contaminant could provide a viable avenue to score increased incidence of mutations.

### 4.2. Gene flow in the pollution cline

Our estimates of migration among populations were conducted using MIGRATE's coalescent framework. As such, the estimates presented here represent gene flow averages over *N*_e_ generations [50]. However, Samarasin *et al.* [51] showed that, because coalescent methods assume stable migration dynamics, gene flow estimates can be biased if recent migration rates have changed drastically. For instance, if population connectivity was high prior to the onset of pollution and drastically declined after exposure to the pollutants, coalescent estimates will probably underestimate historical gene flow over *N*_e_ generations. The opposite would be true if one were to extrapolate recent migration rates from coalescent estimates. Interestingly, Samarasin *et al.* [51] also showed that coalescent estimators retained higher accuracy in the face of recent drastic migration changes relative to disequilibrium-based methods (e.g. [52]).

Here we present coalescent-based migration estimates among populations within the pollution cline and flanking sites (figure 4*c,d*). Our analysis had stronger support for models considering each sampling locality as a discrete deme (relative to panmixis; electronic supplementary material, table S2). Similarly, the analysis also showed that migration rates among the pollution cline are high, including the pollution-sensitive population (HS). However, migration rates relative to the flanking pollution-sensitive sites (MA, SR) are reduced (figure 4*c,d*). Moreover, while all migration rates were asymmetric, there were no overall trends towards immigration or emigration. As mentioned above, it is difficult to extrapolate current gene flow rates (i.e. after the onset of pollution) from coalescent estimates. However, our findings do not capture any striking differences in gene flow between pollution-adapted and pollution-sensitive sites. One possibility is that there is not enough resolution in our data to detect nuanced changes in recent gene flow relative to historical trends. However, our data support that migration is open among all demes in the pollution cline (i.e. fine spatial scale), regardless of pollutant concentration.

### 4.3. Population structure across space and time

Population structure in the pollution cline is patchy, with similar levels of genetic diversity among all habitats. These results are consistent with Reid *et al*.'s [12] model of rapid adaptation to pollutants from standing genetic variation. Since selection has only targeted nuclear loci conferring resistance to pollution, all other unlinked loci are free to segregate among individuals throughout the region. As mtDNA is an independent marker, high levels of variation across mtDNA haplotypes could result in similar level of genetic diversity and low genetic differentiation across populations. However, haplotype frequencies revealed fine-scale structure across the pollution cline (figure 3*c*). These results also showed that haplotype frequencies were correlated to the amount of sediment pollution and pollution sensitivity. While suggestive, our data do not allow us to establish a causal link between mtDNA variation and pollution-driven selection. If pollution-driven selection does act on mtDNA, it is considerably weaker than selection on specific nuclear loci (e.g. [12]).

At broad scales, our analyses showed that IBD was an adequate predictor for mtDNA variation among reference and cline populations. Overall, these results are consistent with *F. heteroclitus*' high habitat fidelity and low dispersal rates [15]. We were surprised by the similarity of MA to the most polluted site (figure 4*a* and table 3), especially because their gene flow estimates were low. Anecdotal evidence from local fishermen suggests that a booming trade of *Fundulus* from NBH and SYC sold in Buzzards' Bay for bait may have been involved. However, a more parsimonious explanation is that the apparent similarity is inflated by the loss of haplotype information in the process of estimating genetic distances (i.e. ignoring the linked nature of SNPs in mtDNA). This explanation is supported by the shared haplotype matrix (figure 2*c*) and MIGRATE, which accounts for inter-SNP dependency and treats the data as haplotypes.

We used microsatellite loci to characterize broad-scale changes in population structure across time. While most of our comparisons did not show statistical significance, the comparison between WI-1999 and NBH-2008 was significant. The spatial distance between the sites combined with drift across approximately three generations may be large enough to explain this observation. Testing this hypothesis would require a larger sampling effort with multiple genetic markers across time points. Overall, the microsatellite data supports stable population structure in NBH across time points.

## 5. Conclusion

Our data do not provide evidence for mtDNA pollution-mediated selection in these populations. To the contrary, mtDNA genetic variation is similar throughout all populations. Moreover, we observe fine- and broad-scale population structure between and within clean and polluted habitats based on haplotype frequencies. These differences are particularly pronounced on the proportion of haplotypes carrying non-synonymous SNPs in the most polluted habitat, NBH. We believe this is consistent with either a reduction in *N*_e_ during rapid adaptation, a rapid population expansion following a pollution-bottleneck, or a case of increased mutation rates due to exposure to pollutants. Finally, our demographic analyses suggest that IBD influences the distribution of mtDNA genetic variation at broad spatial scales. This is consistent with *Fundulus*' high site fidelity and low dispersal. At smaller scales (e.g. within the cline), mtDNA variation is patchy and poorly predicted by either distance among microhabitats, sediment concentrations of PCB_126_ or tolerance to PCB_126_ (LC50).

## Supplementary Material

Suplemental figures and Tables

## Supplementary Material

Microsatellite data

## Data accessibility

Mitochondrial reads are available at NCBI in SRA BioProject ID: PRJNA291654. Microsatellite files are also available as the electronic supplementary material.
