# Supplementary material for: Stable genetic structure and connectivity in pollution-adapted and nearby pollution-sensitive populations of Fundulus heteroclitus
Source: R Soc Open Sci. 2018 May 9;5(5):171532. doi: 10.1098/rsos.171532 (PMC5990737; doi:10.1098/rsos.171532)
Supplement: Suplemental figures and Tables [file rsos171532supp1.pdf]

SUPPLEMENTARY MATERIAL FOR:

# Stable genetic structure and connectivity in pollution-adapted and nearby pollution-sensitive populations of *Fundulus heteroclitus*

Joaquin C. B. Nunez, Leann M. Biancani, Patrick A. Flight, Diane E. Nacci, David M. Rand, Douglas L. Crawford, and Marjorie F. Oleksiak

Figure S1: Stepping stone demographic model used to estimate migration rates among populations. At each step of the chain we tested whether a panmixis model or a model of 2 discrete populations would better fit the data. When the latter was true, we estimated migration rates ($M_{k \to i}$) between pairs of populations indicated by the solid black lines. Dashed lines indicate instances where migration was inferred despite populations not being immediately adjacent to each other. Most of these comparisons were done relative to NBH.

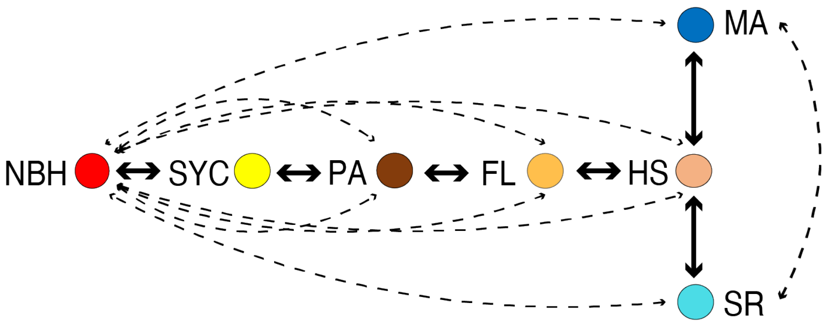

Figure S2: Haplotype PCA done on cline populations. Sediment pollution and LC50 added as supplementary data (green and brown arrows, respectively). The blue arrows correspond to the haplotypes driving the ordination of populations in factor space.

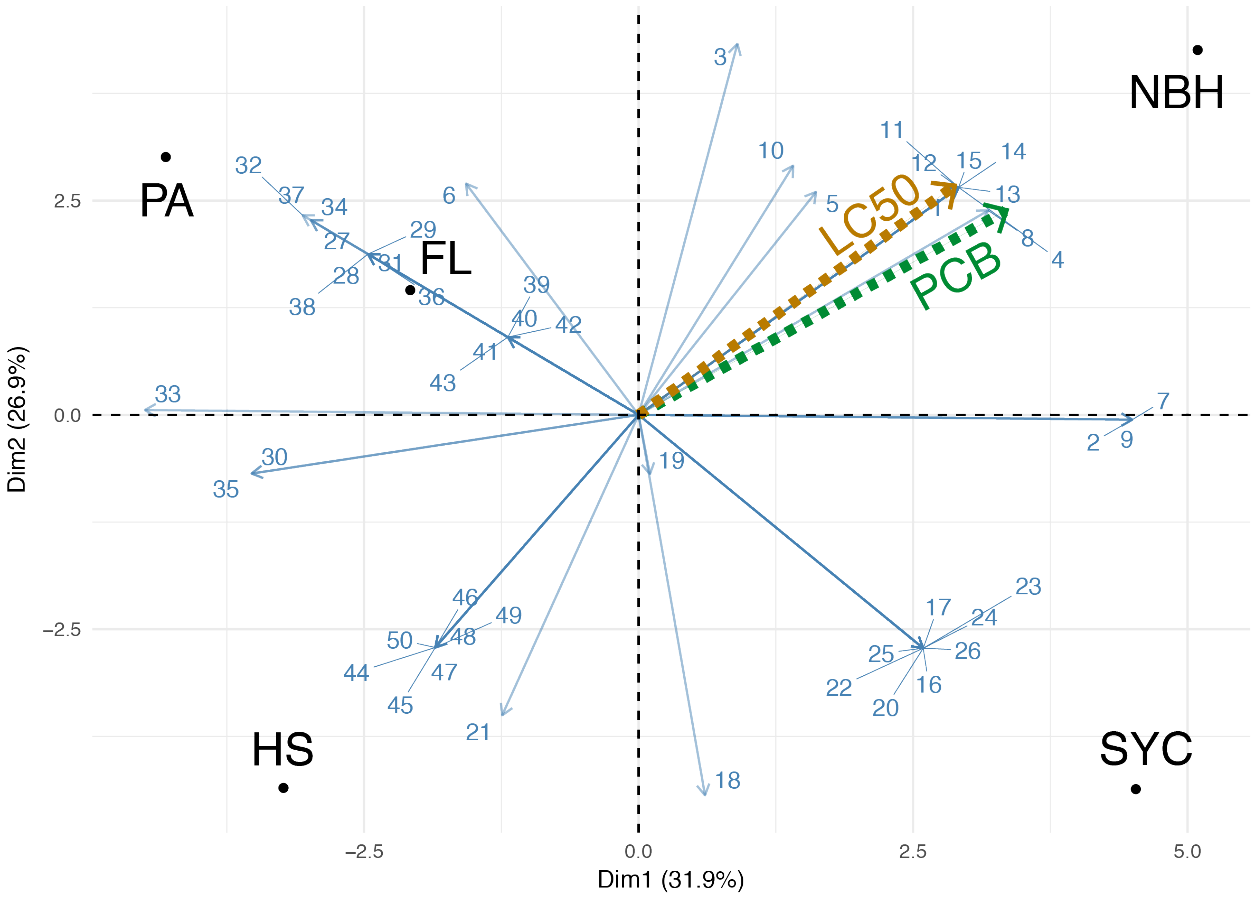

Figure S3: A) Protein topology predictions for mtDNA genes with the locations of non-synonymous SNPs. B) Folding structures of tRNAs and segregating SNPs.

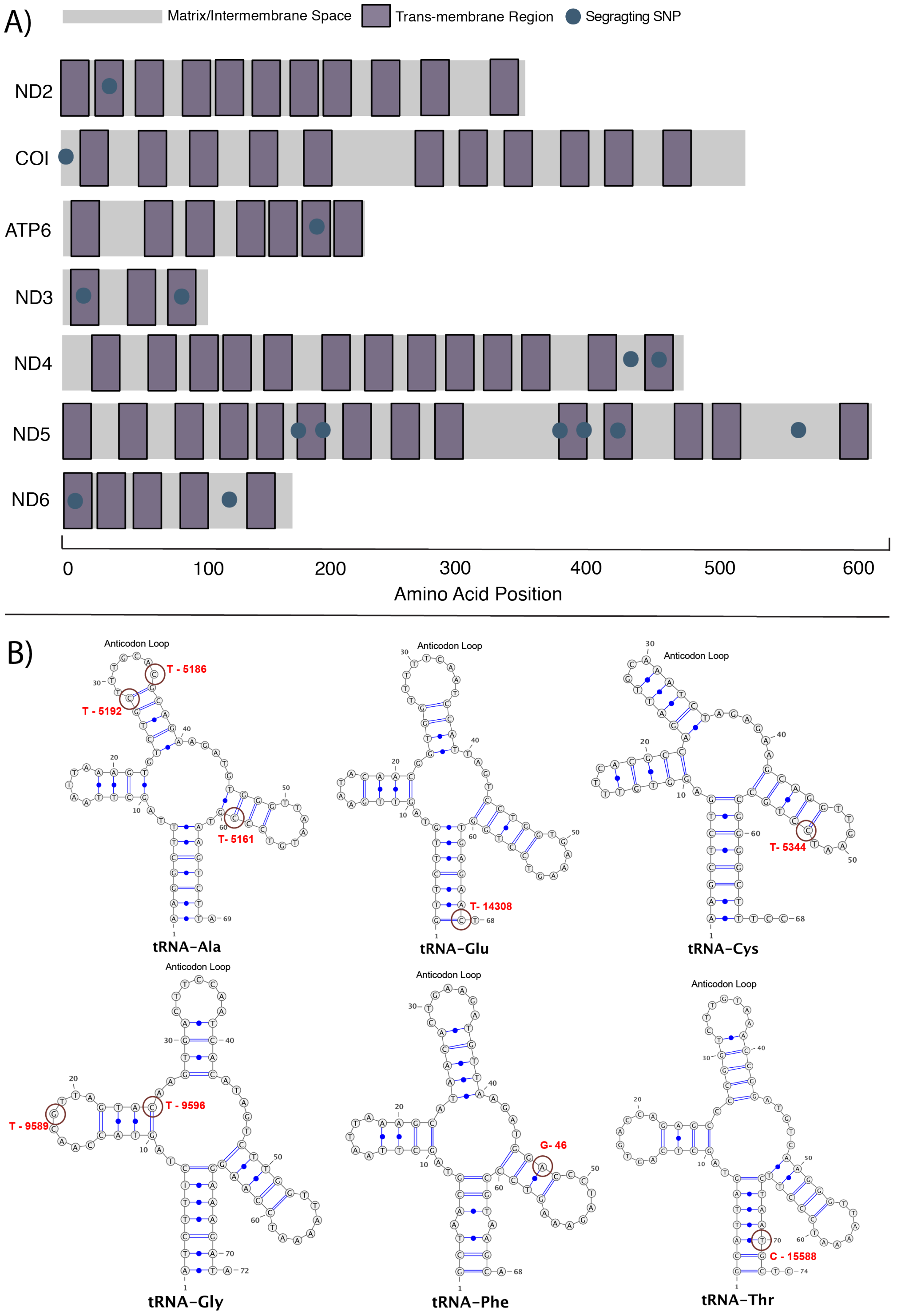

Table S1: Nucleotide diversity at the gene level. Measures of nucleotide diversity (π) *per* protein coding gene, rRNA-12S, rRNA-16s and the D-loop per population. Standard error (SE) is shown in parenthesis.

| Gene | NBH (±SE) | SYC (±SE) | PA (±SE) | FL (±SE) | HS (±SE) | SR (±SE) | MA (±SE) |
|---|---|---|---|---|---|---|---|
| ***ND1*** | 0.055% (0.019%) | 0.084% (0.022%) | 0.101% (0.029%) | 0.118% (0.039%) | 0.090% (0.022%) | 0.024% (0.014%) | 0.054% (0.021%) |
| ***ND2*** | 0.012% (0.011%) | 0.047% (0.022%) | 0.033% (0.016%) | 0.066% (0.028%) | 0.122% (0.028%) | 0.058% (0.020%) | 0.015% (0.013%) |
| ***ND3*** | 0.154% (0.052%) | 0.042% (0.036%) | 0.112% (0.044%) | 0.168% (0.067%) | 0.080% (0.047%) | 0.00% (0.000%) | 0.040% (0.035%) |
| ***ND4*** | 0.074% (0.023%) | 0.023% (0.013%) | 0.086% (0.029%) | 0.107% (0.027%) | 0.075% (0.034%) | 0.041% (0.013%) | 0.011% (0.009%) |
| ***ND4L*** | 0.071% (0.044%) | 0.00% (0.000%) | 0.00% (0.000%) | 0.065% (0.000%) | 0.00% (0.000%) | 0.00% (0.000%) | 0.087% (0.052%) |
| ***ND5*** | 0.187% (0.027%) | 0.134% (0.035%) | 0.086% (0.027%) | 0.054% (0.035%) | 0.029% (0.011%) | 0.034% (0.010%) | 0.149% (0.033%) |
| ***ND6*** | 0.146% (0.029%) | 0.107% (0.030%) | 0.069% (0.021%) | 0.053% (0.032%) | 0.030% (0.025%) | 0.043% (0.035%) | 0.117% (0.029%) |
| ***COI*** | 0.093% (0.018%) | 0.084% (0.022%) | 0.054% (0.014%) | 0.052% (0.017%) | 0.035% (0.013%) | 0.139% (0.020%) | 0.105% (0.024%) |
| ***COII*** | 0.017% (0.015%) | 0. 022% (0.019%) | 0.000% (0. 000%) | 0.000% (0.000 %) | 0.000% (0.000%) | 0.066% (0.026%) | 0.000% (0.000%) |
| ***COIII*** | 0.056% (0.021%) | 0.100% (0.025%) | 0.028% (0.017%) | 0.028% (0.017%) | 0.018% (0.016%) | 0.059% (0.024%) | 0.067% (0.024%) |
| ***ATP6*** | 0.064% (0.026%) | 0.082% (0.031%) | 0.054% (0.028%) | 0.084% (0.046%) | 0.040% (0.024%) | 0.016% (0.015%) | 0.020% (0.017%) |
| ***ATP8*** | 0.000% (0. 000%) | 0.000% (0. 000%) | 0.000% (0. 000%) | 0.000% (0. 000%) | 0.000% (0. 000%) | 0.000% (0. 000%) | 0.000% (0. 000%) |
| ***CytB*** | 0.041% (0. 015%) | 0.105% (0. 023%) | 0.096% (0. 025%) | 0.118% 0. 034%) | 0.061% (0. 021%) | 0.074% (0. 027%) | 0.126% (0. 027%) |
| **rRNA – 12S** | 0.081% 0. 017%) | 0.028% (0. 016%) | 0.037% (0. 014%) | 0.039% (0. 021%) | 0.026% (0. 016%) | 0.085% (0. 017%) | 0.036% (0. 019%) |
| **rRNA – 16S** | 0.013% (0. 008%) | 0.060% (0. 019%) | 0.054% (0. 015%) | 0.045% (0. 014%) | 0.025% (0. 012%) | 0.083% (0. 016%) | 0.050% (0. 021%) |
| **D-Loop** | 0.052% (0. 018%) | 0.075% (0. 024%) | 0.041% (0. 022%) | 0.061% (0. 026%) | 0.085% (0. 024%) | 0.085% (0. 034%) | 0.014% (0. 012%) |

Table S2: Model evidence migration models. For all comparisons 2 models were evaluated: A panmixis model (were the 2 samples are 1 population), and a 2-population model with asymmetrical migration (2-pop-M). Model evidence was determined using log Bayes factors (LBF). Model convergence in MIGRATE is shown by effective sample sizes (ESS). In all cases, the 2-pop-M model had higher support (relative to panmixis).

| Comparison | Model | ln(*P*(D∣Model)) | LBF (relative to panmixis) | ESS |
|---|---|---|---|---|
| **NBH, SYC** | Panmixis | -24654.6 | 0.0 | 3174.3 |
| | 2-pop-M | -24418.3 | 472.7 | 3600.0 |
| **SYC, PA** | Panmixis | -25167.8 | 0.0 | 1677.2 |
| | 2-pop-M | -25005.9 | 323.9 | 1963.5 |
| **PA, FL** | Panmixis | -24863.2 | 0.0 | 3558.9 |
| | 2-pop-M | -24615.7 | 495.1 | 4060.7 |
| **FL, HS** | Panmixis | -24471.8 | 0.0 | 4275.9 |
| | 2-pop-M | -24298.2 | 347.2 | 5485.0 |
| **HS, MA** | Panmixis | -25158.6 | 0.0 | 2433.6 |
| | 2-pop-M | -24955.7 | 405.7 | 3882.3 |
| **HS, SR** | Panmixis | -25248.0 | 0.0 | 2181.6 |
| | 2-pop-M | -25001.1 | 493.9 | 3260.9 |
| **NBH, SR** | Panmixis | -25424.4 | 0.0 | 2320.5 |
| | 2-pop-M | -25149.6 | 549.8 | 3726.1 |
| **NBH, MA** | Panmixis | -25154.5 | 0.0 | 2253.9 |
| | 2-pop-M | -24913.5 | 482.1 | 3205.1 |

Table S3: Amino-changes resulting from segregating SNPs throughout all populations.

| Gene | Strand | Polypeptide Position | Amino-Acid Change |
|---|---|---|---|
| *ND5* | + | 178 | V → A |
| | | 195 | A → V |
| | | 426 | A → T |
| | | 374 | F → L |
| | | 381 | I → T |
| | | 583 | T → A |
| *ND6* | - | 10 | F → L |
| | | 108 | S → N |
| | | 115 | M → T |
| *ND4* | + | 426 | L → F |
| | | 450 | A → T |
| *ND3* | + | 8 | M → I |
| | | 74 | L → V |
| *ND2* | + | 47 | S → Y |
| *ATP6* | + | 188 | M → T |
| *COI* | + | 1 | Initiator codon presumably conserved |

Table S4: SNPs causing changes in non-protein-coding regions of *F. heteroclitus* mtDNA.

| Gene | Strand | Gene Position | Nucleotide Change |
|---|---|---|---|
| 12S - rRNA | + | 365 | C → T |
| | | 489 | T → A |
| | | 570 | T → C |
| 16S - rRNA | + | 66 | T → C |
| | | 247 | A → G |
| | | 1202 | A → G |
| tRNA - Ala | - | 5161 | C → T |
| | | 5186 | C → T |
| | | 5192 | C → T; anticodon loop |
| tRNA - Cys | - | 5344 | C → T |
| tRNA - Glu | - | 14308 | C → T |
| tRNA - Gly | + | 9589 | G → T |
| | | 9596 | C → T |
| tRNA - Phe | + | 47 | G → A |
| tRNA - Thr | + | 15588 | T → C |
